# Assessment of haptoglobin alleles in autism spectrum disorders

**DOI:** 10.1038/s41598-020-64679-w

**Published:** 2020-05-08

**Authors:** Francesca Anna Cupaioli, Ettore Mosca, Chiara Magri, Massimo Gennarelli, Marco Moscatelli, Maria Elisabetta Raggi, Martina Landini, Nadia Galluccio, Laura Villa, Arianna Bonfanti, Alessandra Renieri, Chiara Fallerini, Alessandra Minelli, Anna Marabotti, Luciano Milanesi, Alessio Fasano, Alessandra Mezzelani

**Affiliations:** 1 Institute of Biomedical Technologies, National Research Council, Via Fratelli Cervi 93, 20090 Segrate, Italy; 2 Department of Molecular and Translational Medicine, Biology and Genetic Unit, University of Brescia, 25123 Brescia, Italy; 3 Genetics Unit, IRCCS Istituto Centro S. Giovanni di Dio, Fatebenefratelli, 25123 Brescia, Italy; 4 Scientific Institute, IRCCS Eugenio Medea, Bosisio Parini, Lecco, Italy; 5 Medical Genetics, University of Siena, Siena, Italy; 6 Genetica Medica, Azienda Ospedaliera Universitaria Senese, Siena, Italy; 7 Department Chemistry and Biology, “A. Zambelli”, University of Salerno, Via Giovanni Paolo II 132, 84084 Fisciano, (SA) Italy; 8 Mucosal Immunology and Biology Research Center, Center for Celiac Research and Treatment, and Division of Pediatric Gastroenterology and Nutrition, Massachusetts General Hospital - Harvard Medical School, 55 Fruit Street, Boston, MA 02114 USA; 9 European Biomedical Research Institute of Salerno (EBRIS), EBRIS Foundation, Via Salvatore de Renzi, 3, Salerno, 84125 Italy

**Keywords:** Neuroscience, Risk factors

## Abstract

Gene-environment interactions, by means of abnormal macromolecular intestinal adsorption, is one of the possible causes of autism spectrum disorders (ASD) predominantly in patients with gastrointestinal disorders. Pre-haptoglobin-2 (zonulin), encoded by the *Haptoglobin* (HP) allele-2 gene, enhances the intestinal permeability by modulation of intercellular tight junctions. The two alleles of *HP*, *HP1* and *HP2*, differ for 2 extra exons in *HP2* that result in exon duplication undetectable by classic genome-wide association studies. To evaluate the role of *HP2* in ASD pathogenesis and to set up a method to discriminate *HP* alleles, Italian subjects with ASD (n = 398) and healthy controls (n = 379) were genotyped by PCR analysis; subsequently, the PCR results were integrated with microarray genotypes (Illumina Human Omni 1S-8), obtained using a subset from the same subjects, and then we developed a computational method to predict *HP* alleles. On the contrary to our expectations, there was no association between *HP2* and ASD (P > 0.05), and there was no significant allele association in subjects with ASD with or without gastrointestinal disorders (P > 0.05). With the aid of bioinformatics analysis, from a window frame of ~2 Mb containing 314 SNPs, we obtain imputation accuracy (r^2^) between 0.4 and 0.9 (median 0.7) and correct predictions were between 70% and 100% (median 90%). The conclusions endorse that enhanced intestinal permeability in subjects with ASD should not be imputed to *HP2* but to other members of the zonulin family and/or to environmental factors.

## Introduction

Autism spectrum disorders (ASD) are a group of neurodevelopmental disorders with a complex and heterogeneous etiology that appears within the 3rd year of subjects and affects mainly males (ratio at 8 years of age male:female 4,5:1)^1^. Subjects with ASD are characterized by impaired interpersonal relationship, verbal and non-verbal communication difficulty, restricted and repetitive behavior.

ASD are often associated with comorbidities such as mental retardation, epilepsy, metabolic impairment, autoimmune and gastrointestinal disorders (GIDs) with microbial dysbiosis and enhanced intestinal permeability^2–5^.

Over the last decades, a dramatic increase of the disorder has occurred. This is justifiable to a limited extent, due to changes in diagnostic criteria in the Diagnostic and Statistical Manual of Mental Disorders (DSM) over the years. Genetics play a key role in autism pathogenesis and hundreds of genes have been associated to ASD, only a mere 20–25% of autistic subjects carry a causative genetic variant^6^. A gene-environment interaction has been proposed for the remaining cases, especially in cases with GIDs that correlate with the severity of ASD symptoms^7,8^. GIDs cases are often associated to “leaky gut” and the alteration in normal commensal gut microbiota (dysbiosis)^7^ leading to an increase in inflammation, abnormal macromolecule trafficking, potential translocation of intestinal microorganisms to lamina propria and blood^9^. Furthermore, dysbiosis is coupled with the production of toxins and an adsorption dysfunction. Many environmental factors including intestinal microorganisms (i.e. *Candida albicans*), bacterial toxins such as zonula occludens toxin (Zot) secreted by the pathogen *Vibrio cholerae* as well as food mycotoxins can alter the intestinal permeability^10^. Zot is a well-known toxin that induces severe intestinal permeability, that leads to the reversible opening of intestinal intercellular tight junctions *via* dissociation of Zonula Occludens-1 from its transmembrane binding partners occludin and claudin-1^11^. A human protein called zonulin has been identified and resembles similarities with Zot. The human zonulin, that corresponds to pre-haptoglobin 2 (pre-HP2), shares a common motif with the active fragment of Zot that is critical for intestinal receptor binding. Pre-HP2 increases intestinal permeability in *ex vivo* and *in vivo* murine intestine in a dose-dependent manner, but not mature HP2^12^. For these reasons, the role of human pre-HP2 in modulation of intestinal permeability is suggested due to its similarity with Zot^10,13^ in subjects with *HP2* allele.

Mature HP, is an acute phase protein with a haemoglobin-binding function. Its presence is abundant in plasma where it acts as a free haemoglobin scavenger preventing oxidative damage between heme iron and protein or lipids. It also has an important role as an angiogenic, anti-inflammatory and immune modulator factor^14^. HP is mainly expressed in the liver and its synthesis is induced by cytokines, especially interleukin (IL) -6, IL-1 and tumour necrosis factor (TNF)^15^.

**Figure 1:**
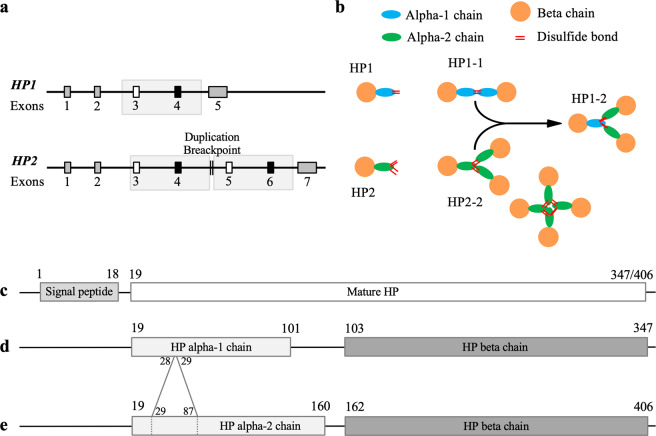
*HP* alleles and protein structures. (**a**) *HP1* and *HP2* allele structure; *HP1* allele contains 5 exons, while *HP2* is made up of 7 exons: exons 1 to 4 correspond to *HP1*, exons 5 and 6 is the duplication of exon 3 and 4, and exon 7 corresponds to exon 5 in *HP1*. (**b**) HP isoform and protein structure. *HP1* allele encode for alpha-1 and beta chain (in blue and orange, respectively), while *HP2* allele for alpha-2 and beta chain (green and orange respectively). The quaternary structures of *HP* genotypes are also illustrated (modified from^17^). (**c**) *HP* gene encode for signal peptide, from amino-acid residues 1–18, and mature protein sequence from 19 to 347 or 406, depending on the allele of origin: *HP1* allele encoding for alpha-1 chain (residues 19–101) and beta chain (**d**), *HP2* allele for alpha-2 chain (residues 19–160) and beta chain (**e**). Alpha-1 chain lacks of residues 29–87 of alpha-2 chain, and beta chain is common to *HP1* and *HP2* alleles.

In humans, HP (UniProt accession number P00738; GI: 386783) is encoded by 2 alleles, *HP1* and *HP2* (Gene ID: 3240)^16^ yielding three different genotypes: HP1-1, HP1-2 and HP2-2. *HP1* and *HP2* alleles differ for the duplication of exons 3 and 4, resulting in exons 5 and 6 in *HP2* (Fig. 1). The precursor protein (pre-HP) includes signal peptide (amino-acids position: 1–18) and mature protein sequences, containing both alpha and beta chains. The proteolitically cleaved beta chain is common in HP from *HP1* and *HP2* alleles, while alpha chain differs from HP1 to HP2: the alpha chain from *HP2* (alpha-2 chain) includes amino-acids at position 19–160, while alpha chain from *HP1* (alpha-1 chain), lacks of residues 29–87 in comparison to alpha-2 chain, and includes residues from 19 to 101 (Fig. 1). The exon 3 contains the protein multimerization domain, allowing quaternary structure formation. Alpha and beta chains combine as tetramer to obtain HP. As a consequence of *HP* allelic variant, the structure of HP in HP1-1 phenotype is (alpha-1-beta)_2_, that of HP1-2 are (alpha-1-beta)_2_ and (alpha-2-beta)_n_, and that of HP2-2 is (alpha-2-beta)_n_^17,18^ (Fig. 1). HP1, on the other hand, displays fast (HP1F) and slow (HP1S) isoforms of the alpha chain, depend on their fast (F) or slow (S) electrophoretic motilities. These two forms differ for the presence of an amino-acid substitution (Lys54 of HP1S is replaced by Glu in the HP1F)^19^ and originate from different allelic subtypes: *HP1F, HP1S, HP2FF, HP2FS* and *HP2SS* (very rare). Because of the structure of the two alleles, it is very difficult to recognize them by means of classic genome-wide association studies (GWAS), so most of the genotyping studies are limited to small size populations analyzed by PCR or quantitative RT-PCR.

HP1 and HP2 proteins are functionally different. HP1-1 protein has a superior antioxidant capacity than HP2-2 and induces a more rapid free hemoglobin degradation^17,20^, *HP1* carriers also have a higher HP concentration than *HP2*, with HP1-1 > HP1-2 > HP2-2^21^.

Due to the protective role of HP1*, HP1-1* genotype has a significant association to longevity^22^, on the other hand, *HP2-2* has a higher susceptibility to immune-mediated diseases, such as celiac disease, Rheumatoid Arthritis, Type 1 diabetes, Systemic Lupus Erythematosus^23–26^ and cancer.

In 2000, Wang and collaborators reported that an 8 amino-acid sequence in N-terminal of human pre-HP2 (residues 8–15: G G V L V Q P G) shared a common motif with the active fragment of Zot (residues 291-298-: G R L C V Q D G) from which originated the alternative name “zonulin”. The common amino-acid motif is: non-polar (G), variable, non-polar, variable, non-polar (V), polar (Q), variable, non-polar (G)^13^. In 2018, Scheffler and collaborators identified another member of the zonulin family, properdin, a mannose-associated serine protease^27^.

Zonulin induces the disassembling of intercellular tight junctions in many epithelial and endothelial barriers, including the “leaky gut” and enhancing the intestinal trafficking of microorganisms and macromolecules that, in sequence, challenge the immune response involving different tissues and organs in genetically susceptible subjects^12,26,28^.

Of interest, a significant high level of zonulin (pre-HP2) in a small cohort of subjects with ASD *vs* healthy controls was found^29^, as well as an association between *HP2* allele and autism with GID comorbidity^30^.

In this study, we genotyped a larger cohort of patients and controls to test the hypothesis that *HP2* allele is associated with ASD development at least with GID in autism. We assessed the *HP* alleles in a batch of Italian subjects with ASD (n = 398) and healthy controls (n = 380) by PCR analysis. Because of the internal exon duplication of *HP*, it is very difficult to distinguish the two different alleles by means of standard bioinformatics analysis, and PCR techniques limit the size of tested subjects. We integrated PCR and microarray genotyping data, previously obtained on a subgroup of patients and controls, to impute and discriminate *HP* alleles by GWAS. Thus, we provide a bioinformatic reference useful for further *HP* prediction on publicly available GWAS data.

## Materials and Methods

### Ethics committees

- Ethics committee IRCCS Eugenio Medea – Scientific section of the association “La Nostra Famiglia” 2010, IRCCS Eugenio Medea – La Nostra Famiglia, Bosisio Parini (LC), Italy;
- Ethics committee of the hospital institutions (CEIOC) IRCCS Istituto Centro San Giovanni di Dio Fatebenefratelli 50/2008, IRCCS Istituto Centro S. Giovanni di Dio Fatebenefratelli, Brescia, Italy;
- Local ethics committee, Prot. 567/06 dated 28-09-2006 and document integration dated 09-10-2006, Azienda Ospedaliera Universitaria Senese, Siena, Italy.

All Ethics Committees of involved Institutions approved the study and enrolled subjects that signed informed consents. Informed signed consents were collected from parents and/or legal guardians for subjects under the age of 18 years.

All methods were performed in accordance with relevant guidelines and regulations regarding observational studies.

### Subjects

A total sample-set consisting of 398 Italian subjects with ASD [338 males (85%) and 60 females (15%)], and 379 unrelated Italian controls [170 males (45%) and 209 females (55%)] were recruited and peripheral blood samples collected.

### Patients

IRCCS Eugenio Medea – La Nostra Famiglia recruited 206 subjects with ASD [171 males (83%) and 35 females (14%)] ranging between 2 and 12 years old. The diagnosis for autism (70.3%), Asperger’s syndrome (3.5%) or pervasive developmental disorder not otherwise specified (26.2%) were performed according to DSM, fourth edition, text revision (DSM-IV TR) (American Psychiatric Association [APA], 2000) and by the Autism Diagnostic Observation Scale 2 (ADOS-2)^31^ and Autism Diagnostic Interview - Revised (ADI-R)^32^. Subjects with genetic syndromes, epilepsy and neuroradiological confirmed disorders were excluded. Clinical data about the presence/absence of GIDs were evaluated in a sub-group of 157 subjects with ASD [135 males (86%) and 22 females (14%)].

The University of Siena recruited 192 subjects with ASD [167 males (87%) and 25 females (13%)] ranging from 1 to 3 years old. The diagnosis for autism (82%), Asperger’s syndrome (0,5%) and pervasive developmental disorders not otherwise specified (17%) were performed according to the DSM-IV TR (APA, 2000), and by the ADOS-2 and ADI-R. Within these patients and clinical data about the presence/absence of GIDs were evaluated in a sub-group of 8 male subjects.

### Controls

IRCCS Eugenio Medea – La Nostra Famiglia recruited 25 unrelated children (12 males and 13 females) evaluated by Child Behavior Checklist as neurotypical controls; the University of Brescia and IRCCS Istituto Centro S. Giovanni di Dio, Fatebenefratelli of Brescia enrolled 259 healthy subjects [109 males (42%) and 150 females (58%); mean age 51.6 ± 15.5]; 191 (73.7%) were unrelated non-affected volunteers who were screened for DSM-IV Axis I disorders by expert psychologists using the Mini-International Neuropsychiatric Interview^33^. Only subjects without a history of drug or alcohol abuse/dependence and without a personal or first-degree family history of psychiatric disorders were enrolled in this study. Furthermore, subjects who obtained a score lower than 27/30 at the Mini Mental State Examination^34^ were excluded. The University of Siena recruited 95 healthy controls [45 females (47%) and 50 males (53%), aged between 20 years and 61 years, mean age of 41 years]. The subjects were healthy adults who were not evaluated for neuropsychiatric conditions.

### DNA isolation

Genomic DNA was extracted from peripheral blood leukocytes, using commercial kits and relative protocols. Quality (280/260 ratio) and quantity of DNA was checked by NanoDrop 2000 spectrophotometer (Thermo Fisher Scientific, Wilmington, DE USA).

### Haptoglobin genotyping

PCR - *HP *genotypes were determined by PCR amplification devised by Koch and collaborators^35^ with minor modifications. Briefly, new PCR primers (Table 1) were designed on *HP1* and *HP2* specific sequences M69197.1 (EMBL-GeneBank Data Libraries) and 30 ng of genomic DNA was amplified in 10 μl of reaction mixture (Platinum™ SuperFi™ PCR Master Mix, for primers NewA and NewB, and Platinum® PCR SuperMix, High Fidelity, for primers NewC and NewD- Invitrogen™, Thermo Fisher Scientific, Wilmington, DE USA) as suggested by the supplier. Two couples of primers, NewA with NewB and NewC with NewD were used separately, to analyze every sample and each analysis was duplicated. Primers NewA (5′- GGGGTTCCTGCCAGAAATGA -3′) and NewB (5′- CCCTGGCTGGTGAACTGTATT -3′) can produce, contextually, a 1775 bp and/or a 3487 bp specific product for *HP1* and *HP2* alleles, respectively. Primers NewC (5′- ATGCCAACCTGCCTCGTATT -3′) and NewD (5′- CGAACCGAGTGCTCCACATA -3′) amplify a 360 bp *HP2* allele specific fragment.

**Table 1:** PCR primers sequence and amplicon length.

Primer	Primer sequence	Amplicon length	
		HP-1	HP-2
NewA	5′- GGGGTTCCTGCCAGAAATGA -3′	1775 bp	3487 bp
NewB	5′- CCCTGGCTGGTGAACTGTATT -3′		
NewC	5′- ATGCCAACCTGCCTCGTATT -3′	—	360 bp
NewD	5′- CGAACCGAGTGCTCCACATA -3′		

**Figure 2:**
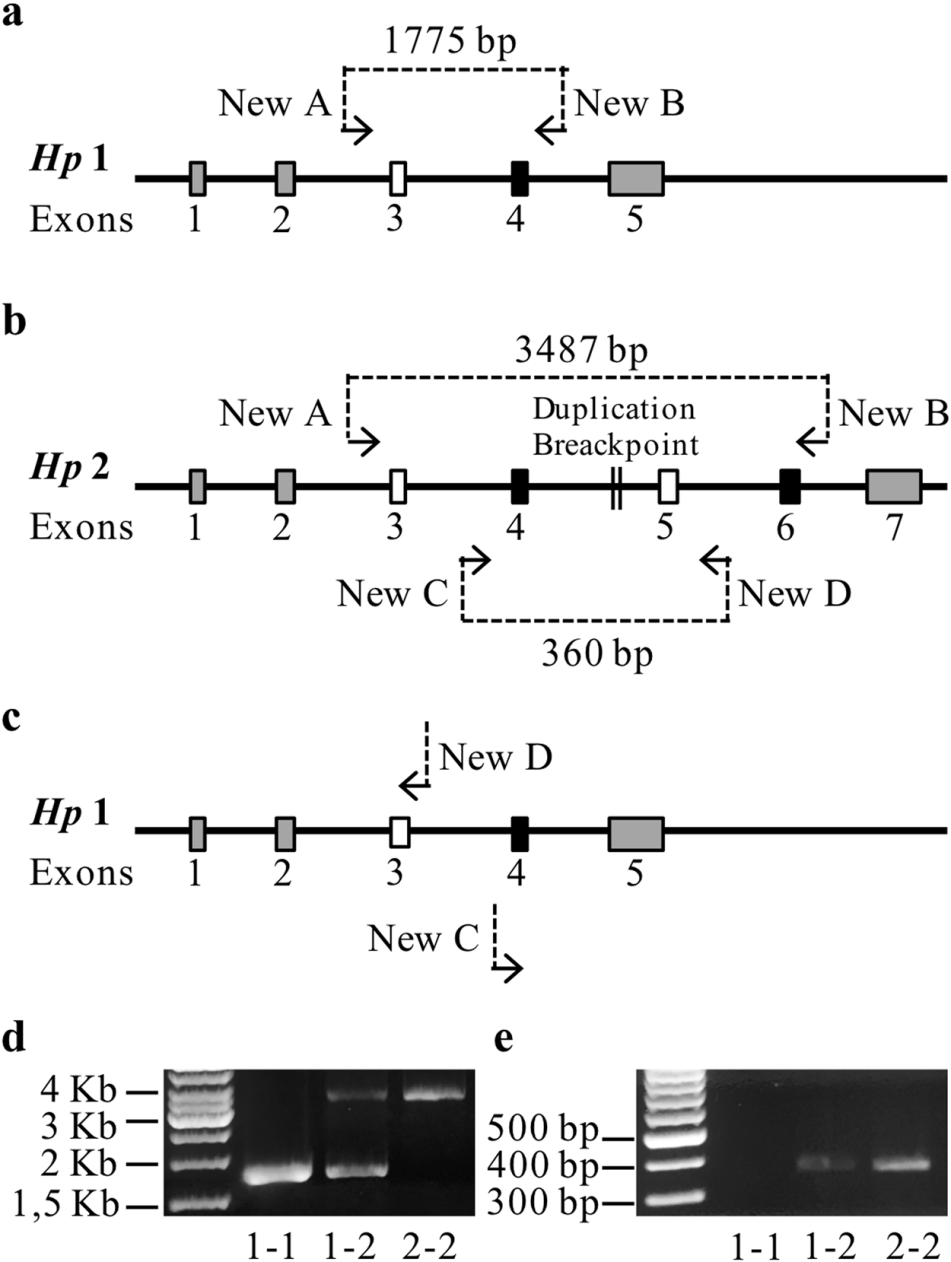
*HP* alleles structure and PCR primers. *HP2* allele contains a duplication of 1700 bp, corresponding to extra exons 3 and 4. Due to this duplication, PCR amplification with NewA and NewB primers (narrowly indicate 5′ > 3′ orientation) amplification gives 1775 bp length amplicons in HP1 allele (**a**), and 3487 bp length amplicons in *HP2* allele (**b**). PCR products obtained with primers pairs NewC/NewD are 360 bp long in *HP2* allele (**b**), while in *HP1* no amplicon is given (**c**). Agarose gels, 1% (**d**) and 2% (**e**), are us**e**d to detect the three different genotype for both primer pairs. In the case of NewA and NewB in HP1-1 subjects only 1775 bp amplicon is present, in HP1-2 both amplicons are present and in HP2-2 only 3487 bp amplicon is detected (**d**). For NewC/NewD primer pairs 360 bp amplicons is detected only in HP1-2 and HP2-2 genotypes (**e**). Gels images (**d**,**e**) are cropp**e**d from images of full-length gels (Supplementary Fig. S1).

After denaturation for 30 seconds at 98 °C, thermo-cycling profile consisted of denaturation for 12 seconds at 98 °C, annealing for 10 seconds at 65.4 °C and extension for 1 minute and 40 seconds at 72 °C repeated for 35 cycles when using primers NewA and NewB, while denaturation for 2 minutes at 94 °C, thermo-cycling profile for 1 minutes at 94 °C, 40 seconds at 68 °C and 50 seconds at 72 °C repeated for 32 cycles, and extension for 2 minutes at 72 °C for primers NewC and NewD. PCR products obtained by NewA-B and NewC-D couples of primers were run on 1% and 2% agarose gel (E-Gel agarose gels, Invitrogen™, Thermo Fisher Scientific) respectively. In HP1-1 we detected the NewA-B PCR product of 1775 bp length, but not NewC-D PCR product; in HP2-2 we found the NewA-B PCR product of 3487 bp and NewC-D 360 bp PCR product; in HP1-2 subjects we identified both 3487 bp and 1775 bp amplicons, and the 360 bp PCR product (Fig. 2).

### Sanger sequencing

To check the PCR products, two PCR products for each type of *HP* genotype obtained by NewA and –B amplification (HP1-1, HP1-2, HP2-2) as well as those obtained by NewC and -D, were purified (CleanSweep PCR Purification Reagent, Applied Biosystems, Thermo Fisher Scientific, Wilmington, DE USA) and sequenced by Sanger method using NewC and NewD respectively for forward and reverse sequencing. Results were compared to *HP1* and *HP2* sequences (NCBI Reference Sequence: NG_012651.1).

### Data analysis

The association between *HP* alleles and ASD was calculated considering subjects with ASD *vs* all the analyzed controls and subjects with ASD without GID *vs* subjects with ASD with GID. Chi-square with Yates correction test was applied to evaluate statistical significance. We sub-classified the controls in two groups referring to the enrollment screening test: ASDs negative controls (n = 188), referred as non-ASD (NASD) controls, and those negative for ASD and DSM-IV Axis I disorders (n = 191), referred as super-controls. The *HP* allelic distribution was evaluated in all subjects with ASD and controls and in all sub-groups. Possible effect of sex on HP allele distribution was investigated in patients with ASD and in healthy subjects, considering the sub-classification of controls.

Due to the small size of controls enrolled in this study we implemented our data by a meta-analysis including *HP* genotyping of healthy Italians^22,36^, healthy Caucasians^35^ (Table 2).

**Table 2:** *HP* allelic distribution in subjects with ASD and controls.

			Controls enrolled in this study			Controls from literature				Subjects with ASD without GID	Subjects with ASD with GID
		Subjects with ASD	Total	Super controls	NASD control	Neurotypical Italian populations	Total controls + neurotypical Italian population	NASD controls + neurotypical Italian populations	Caucasian healthy subjects		
		n = 398	n = 379	n = 191	n = 188	n = 2130	n = 2509	n = 2318	n = 249	n = 76	n = 89
Genotype	**HP1-1**	59 (14.8%)	35 (9.2%)	18 (9.4%)	17 (9.1%)	294 (13.8%)	329 (13.1%)	311 (13.4%)	36 (14.5%)	13 (18.1%)	12 (14.1%)
	**HP1-2**	175 (44.0%)	163 (43.0%)	68 (35.6%)	95 (50.5%)	913 (42.9%)	1076 (42.9%)	1008 (43.5%)	120 (48.2%)	25 (34.7%)	39 (45.9%)
	**HP2-2**	164 (41.2%)	181 (47.8%)	105 (55.0%)	76 (40.4%)	923 (43.3%)	1104 (44.0%)	999 (43.1%)	93 (37.3%)	34 (47.2%)	34 (40.0%)
Allele frequency	***HP1***	293 (36.8%)	233 (30.7%)	104 (27.2%)	129 (34.3%)	1501 (35.2%)	1734 (34.6%)	1630 (35.2%)	192 (38.6%)	51 (35.4%)	63 (37.1%)
	***HP2***	503 (63.2%)	525 (69.3%)	278 (72.3%)	247 (65.7%)	2759 (64.8%)	3284 (65.4%)	3006 (64.8%)	306 (61.4%)	93 (64.6%)	107 (62.9%)
	**P value**		0.0134*	0.0014**	0.4429	0.4169	0.2480	0.3904	0.5673		0.8542

Data on neurotypical Italian population were from the total of controls enrolled by Bottini and collaborators and Napolioni and co-worker^22,36^. Genotyping data on Caucasian healthy subjects were from Koch and collaborators^35^, and *HP* allele frequency of individuals sampled by the 1000 Genomes Project was calculated in the study of Boettger and co-worker^58^. *HP2* allele is highly represented in subjects with ASD and controls. *HP1* allele increases significantly in patients when compared to total and non-ASDs controls enrolled in this study and to controls from 1000 Genomes projects. In contrast, *HP1* allele did not increase significantly when compared to the other controls. P value was calculated with Chi-square with Yates correction test (*P < 0.05; **P < 0.005).

### Microarray

Microarray analysis was performed on a subset of samples (n = 318) collected by IRCCS Eugenio Medea – La Nostra Famiglia using Human Omni 1S-8 v 1.0 Illumina chip and iScan Illumina (Illumina, San Diego, CA, USA) according to the manufacturer’s protocols, and a selection of 230 subjects were genotyped by microarray and PCR.

Among controls collected by the University of Brescia, 177 subjects (87 females and 90 males) were genotyped by Affymetrix Human Mapping GeneChip 6.0 array (Affymetrix, Thermo Fisher Scientific, Wilmington, DE USA) according to the manufacturer’s protocols and applying quality control procedures described by Sacchetti and colleagues^37^, and 103 of them were genotyped for HP gene by PCR.

### Bioinformatics

Genetic data for 1,185,076 SNPs in 230 subjects were collected from “Top” alleles of the Illumina’s Genome Studio Final Report, generated using HumanOmni1S platform and GRCh37.p13 genome assembly. Standard quality control procedures were used to exclude individuals with discordant sex and call rates below 98% and filter out SNPs with MAF <1%, Hardy Weinberg *p* < 1 × 10^−4^ and a call rate <99%. A total of 696,849 markers and 293 subjects fulfill quality control requirements. The analysis of genetic similarity among individuals revealed the presence of several outliers. Only individuals belonging to the most populated cluster were considered for further analyses. The quality control procedures repeated on this subgroup determined the exclusion of some additional markers. A total of 690’215 markers and 282 individuals were retained.

Genotypes detected by microarray and *HP* genotypes detected by PCR were phased using Beagle^38^ against the haplotypes provided by the 1000 Genomes project (Phase 3). *HP* alleles were imputed using Beagle v4.1^39^. A total of 100 cross-validation trials were run, randomly assigning each time 90% of the subjects to the “reference” group and the remaining 10% to the “test” group.

## Results

### HP allele distribution in patients and controls

As for PCR HP genotyping the length of NewA-B PCR products reflected the expected lengths (*HP1* = 1775 bp; *HP2* = 3487 bp) and included the duplication breakpoint sequence only in NewC-D products (360 bp) and in NewA-B products corresponding to *HP2* allele (Fig. 2).

*HP* genotypes of the 398 subjects with ASD were HP1-1 = 59 (14.8%), HP1-2 = 175 (44.0%) and HP2-2 = 164 (41.2%), and the frequencies of *HP1* and *HP2* were 293 (36.8%) and 525 (63.2%), respectively. The distribution of genotype frequencies is in Hardy Weinberg equilibrium (p ≈ 0.3; χ^2^ test).

The genotypes of all the controls (n = 379) were: HP1-1 = 35 (9.2%), HP1-2 = 163 (43.0%) and HP2-2 = 181 (47.8%); *HP1* frequency = 233 (30.7%) and *HP2* = 525 (69.3%); and are in Hardy Weinberg equilibrium (p ≈ 0.9; χ^2^ test). Considering the two control groups separately, the allelic distribution of *HP1* was 34,3% in NASD controls and decreased to 27,2% in super-controls, while *HP2* was 65,7% and 72,3%, respectively (Table 2).

The *HP1* increased in subjects with ASD when compared to the total number of controls: Chi-square with Yates correction test was calculated and a significant increase of *HP1* allele frequency was found in subjects with ASD compared to total controls (NASD controls+super controls) (P < 0.05) and super-controls (P < 0,005). Consequently, the association between *HP1* and ASD disappeared (P > 0.05) excluding super-controls and adding to NASD controls, Italian and Caucasian healthy subjects^22,35,36^ (Table 2).

Considering only subjects with ASD with clinical GID data (n = 157), the allelic distribution in subjects with ASD with GID (n = 89; *HP1* 37.1% and *HP2* 62.9%) and without GID (n = 76; *HP1* 35.4% and *HP2* 64.6%) did not differ (P > 0.05) (Table 2).

The sex ratio differs from the subjects with ASD and controls, and this could influence the *HP* allelic distribution. For this reason, we also investigated *HP* genotype related to sex. *HP* allelic distribution did not show statistically significant imbalanced sex ratio in patients with ASD (P > 0.1), in the total number of controls (P > 0.5), and in the sub-groups NASD controls and super controls (P > 0.1) (Supplementary Table S1). To know more about the consequence of the sex imbalance within controls and subjects with ASD we also re-analyzed data from the study of Brackenridge^40^ that found a significant sex imbalance in HP1-2 distribution within Australian population. *HP* allelic distribution was calculated for healthy Australian population (Supplementary Table S1) and we did not find significant difference in *HP* distribution between males and females by Fisher test (P > 0.5).

### HP alleles imputation from SNP haplotypes

To assess whether *HP* alleles can be predicted by the haplotype of surrounding SNPs we performed a series of cross-validation trials, in which we split the autistic population genotyped with the Illumina array into two groups (“reference” and “test”) panels, and imputed (Beagle v4.1) the *HP* alleles of test subjects, using the multi-SNP haplotypes surrounding *HP* and *HP* alleles detected by PCR in subjects assigned to the reference group. We considered the SNPs occurring up to 2MB around *HP* gene and found the highest accuracy for HP2-2 (0.97) using 79 SNPs (0.5 Mb window). Among *HP* genotypes, we found that genotype 1-1 was the most difficult to predict (Table 3).

**Table 3:** Bioinformatics imputation analysis, Illumina platform.

Window (Mb)	Number of markers	Accuracy of prediction			
		HP1-1	HP1-2	HP2-2	Mean
**0,1**	16	0.34	0.63	0.96	0.73
**0,25**	51	0.70	0.93	0.96	0.91
**0,5**	**79**	**0.80**	**0.92**	**0.97**	**0.92**
**1**	124	0.73	0.94	0.96	0.91
**1,5**	203	0.76	0.93	0.98	0.92
**2**	313	0.79	0.92	0.97	0.92

Results of *HP* alleles imputation from SNP haplotypes genotyped using an Illumina array. Best prediction accuracy results are achieved in increments of 0,5 windows (in bold).

The same approach was applied to healthy subjects, genotyped by the Affymetrix platform (Table 4). We observed a slightly lower accuracy (e.g. 0.89 using 76 SNPs; 0.5 Mb window), but its trends relative to the number of surrounding SNPs considered and to *HP* genotypes were similar to those observed in autistic subjects.

**Table 4:** Bioinformatics imputation analysis, Affymetrix platform.

Window (Mb)	Number of markers	Accuracy of prediction			
		HP1-1	HP1-2	HP2-2	Mean
**0,1**	11	0.26	0.38	0.94	0.67
**0,25**	40	0.64	0.87	0.97	0.89
**0,5**	**76**	**0.59**	**0.89**	**0.97**	**0.89**
**1**	126	0.68	0.90	0.95	0.89
**1,5**	185	0.61	0.85	0.95	0.87
**2**	256	0.67	0.89	0.96	0.90

Results of *HP* alleles imputation from SNP haplotypes genotyped using an Affymetrix platform. Best prediction accuracy results are achieved in increments of 0,5 windows (in bold).

## Discussion

Wang and colleagues first found, in human intestinal tissue, a protein that they called zonulin, sharing a common amino-acid motif with Zot, the *Vibrio cholera* toxin acting on the intestinal wall disassembles tight junctions with consequent intestinal permeability^13^. A following study identified human zonulin, as pre-HP2^12^. Since an involvement of zonulin has already been demonstrated in many immune mediated diseases with GID^41^, and many subjects with ASD suffer from GID and “leaky gut”, a possible role of zonulin in ASD pathogenesis was assumed^29^. Nevertheless, because of its structure containing a duplication of 2 exons, *HP* gene is difficult to genotype by classic genome-wide studies and *HP* available data are limited to a very small amount of cases.

To understand the role of zonulin in ASD etiology and to provide a bioinformatics method to genotype *HP* using or re-using newly generated or archival data, respectively, we *i)* analyzed, by PCR, the *HP* allele distribution in a cohort of Italian subjects with ASD and healthy controls; *ii)* integrated PCR and microarray data of a subgroup of cases, to impute *HP* alleles from flanking SNP haplotypes and discriminate the two *HP* alleles.

Supposing that pre-HP2 corresponds to zonulin, we expected a very significant increase of *HP2* allele frequency in subjects with ASD, or, at least, in cases suffering from GID. What we found is that *HP2* allele prevailed in both subjects with ASD and controls. However, on the contrary to what we expected, there was a decrease in *HP2* frequency when compared to the total number of controls (P < 0.05) in the observed subjects with ASD. Interestingly, this was due to a relative increase of *HP2* in the super-controls rather than a decrease of *HP2* in subjects with ASD. Indeed, comparing *HP* allelic distribution among different Italian control cohorts we found super-controls show a significant increase of *HP2*: P < 0.05 when compared to NASD and P < 0.005 when compared to those of previous study of Bottini and collaborators and Napolioni and co-worker^22,36^. Consequently, the association between *HP2* and ASD disappears when the super-controls are excluded from the data analysis and decreases when comparing the ASDs only with the super-controls (Table 2).

NASD controls were unrelated ASD negative while super-controls belong to a group of a “selected healthy population”, resulting negative when screened for any mental condition (DSM-IV Axis I disorders). In the group of super-controls *HP* alleles frequency was significantly different from that of Italian and Caucasian healthy subjects^22,35,36^ (P = 0.0020 and 0.0006, respectively), while allelic distribution of NASD controls was similar to that reported in literature. This suggests that super-controls are a niche within unrelated non-affected population, therefore it could not be representative for the NASD population. Furthermore, *HP* genotypes distribution within worldwide population differs upon ethnicity and geographical area^42^. For these reasons, *HP* genotype data from previous studies on Italian population^22,36^ were included among NASD controls. No significant differences of allelic distribution were found between ASDs and NASD controls + neurotypical Italian populations. One limitation of this study is the sex-ratio imbalance between controls and subjects with ASD, with an equal distribution of males and females in controls and a prevalence of males in patients. Literature on sex distribution of HP allele within healthy population showed no significant difference in sex-based *HP* allele distribution^40,43^. Indeed, the study of Zhao and colleagues declared no significant sex-based difference in the allelic distribution of *HP*; then again, the re-analysis of the data reported by Brackenridge showing that HP1-2 has different distribution between Australian males and females, revealed no significant differences in *HP* allelic distribution by Fisher test (P = 0.9066) (Supplementary Table S1). To better elucidate this important imbalance in our groups, we investigated the sex-based *HP* allelic distribution within subjects with ASD and controls to solve this bias. No differences were found between *HP* alleles in males and females in patients and total control, neither, separately, in NASD controls or in super controls. Taking together these findings, we conclude that *HP* allelic distribution is not sex dependent and that in these investigations the *HP* allele distribution is not related to sex imbalance between controls and patients with ASD.

A different age distribution between controls (adults) and patients (children) also exists. However, two previous study showed no significant association between age and *HP* genotype in healthy subjects^40,43^.

Moreover, no allelic association was found between subjects with ASD and patients suffering from GID. This indicates that the *HP* genotype does not represent a risk factor for ASDs pathogenesis, and that HP does not have a key role in the onset of GID in subjects with ASD. Furthermore, the significant decrease of *HP1* allele in super-controls should be further investigated.

Our results do not reflect those of two previous studies that analyzed and found a relationship between zonulin and ASD. Indeed, Esnafoglu and collaborators^29^ observed a statistically significant increase of serum zonulin in a group of 32 subjects with ASD compared with 33 healthy controls, by ELISA (Elabscience). Then again, *HP* genotype was determined in a cohort of 46 subjects with ASD (20 of which with GIDs and 26 without GIDs) and 41 controls (6 of which with GIDs and 35 without GIDs) by plasma immunoblot. This study also found an association between HP2-2 and ASD with GIDs when compared with neurotypical developing children (P < 0,01), and on the other hand found no association comparing ASD with GID with ASD^30^. However, these results were obtained from a very small cohort.

Considering the unexpected results of *HP* allele frequency and ASD, we have made some considerations based on literature and the zonulin ELISA commercial kit. Zonulin is often and wrongly considered the alternative name of HP. Indeed, many authoritative databases, including UniProtKB [(P00738 (HPT_HUMAN)], Nextprot (NX_P00738) and NCBI (Gene ID 3234), report both HP and zonulin among “names”, while only pre-HP2 belongs to the zonulin family. This has induced some authors to consider zonulin and HP as the same protein. Furthermore, the zonulin ELISA commercial kits and antibodies also reported indifferently HP or zonulin [i.e. Human Zonulin ELISA Kit, Elabscience, (Wuhan, Hubei Province, China) and IDK® Zonulin ELISA (Immundiagnostik AG, Germany)], and only after the publication of the study of Scheffler and colleagues, the datasheets were corrected reporting zonulin or HP as target protein.

**Figure 3:**
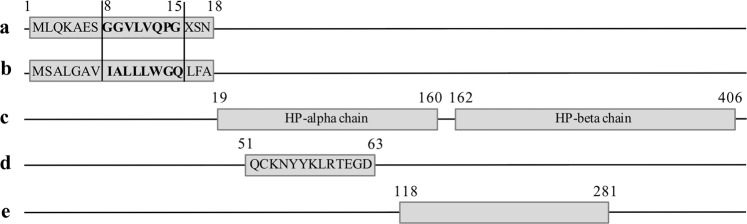
HP protein structure and keynote sequences. (**a**) Signal peptide according to Wang and colleagues^13^; (**b**) Signal peptide from UniProt accession number P00738; (**c**) Mature protein sequence from *HP2* allele made up of alpha and beta chains; (**d**) Sigma-Aldrich declared immunogen sequence; (**e**) Sequence including capture antibody sequence of Human Zonulin ELISA Kit, Elabscience.

Considering zonulin a family of proteins instead of a single one^27^ modifies the interpretation of some previous literature. Indeed, zonulin quantification using commercial Human Zonulin ELISA kits was widely reported in different pathological conditions and the conclusions drawn on the basis of HP-zonulin identity. For instance, Esnafoglu and colleagues used the Human Zonulin ELISA Kit, Elabscience^29^ to quantify zonulin in the serum of subjects with ASD. The capture antibody of this kit recognizes a sequence within residues 118–281 of pre-HP2/zonulin (uniprot ID: P00738) and the detection antibody within residues 104–346 (as referred by technical support, but not reported in the datasheet https://www.elabscience.com/PDF/Cate61/E-EL-H5560-Elabscience.pdf). These sequences may include both alpha (not specified if alpha-1 or alpha-2) and beta chain of HP, and is not specific for the signal peptide characterizing pre-HP2 (Fig. 3). Then again, the capture antibody of the IDK® Zonulin ELISA, recognizes a portion of zonulin previously reported by Wang and colleagues^13^, (datasheet http://www.immundiagnostik.com/fileadmin/pdf/Zonulin_K5601.pdf). Since this portion is not included in HP sequence, the antibody may recognize another zonulin family member, as suggested by Scheffler and colleagues^27^. Indeed, these authors, which integrated antibody capture experiments, mass spectrometry, and Western blot analysis, demonstrated that the IDK® Zonulin ELISA (based on a polyclonal antibody anti-zonulin) mainly recognize properdin, a potential second member of the zonulin family^27^. Interestingly, properdin (P27918 https://www.uniprot.org/uniprot/P27918) maps on Xp11.23 making male subjects more susceptible to its allelic or variant effect. Furthermore, this chromosome region includes three other genes related to autism: the calcium channel, voltage-dependent, alpha 1 F (CACNA1F) associated with syndromic autism and schizophrenia^44^, the phosphatase 1, regulatory (inhibitor) subunit 3 F (PPP1R3F) from which rare mutations have been found in autism^45^ and histone deacetylase 6 (HDAC6) from which a partial skipping of exon 3 was found in a subject with ASD^46^.

Then again, gut dysbiosis has been described in subjects with ASD^47–50^ and gut microbial imbalance, that increases inflammation and microbial toxic metabolites production, is the strongest stimulus for the activation of the zonulin pathway with consequent intestinal permeability and trafficking of macromolecules through intestinal wall^51^. Moreover, gut microbes strongly participate in “microbiota-gut-brain-axis” (the microbiota-CNS cross-talk)^52^ modulating inflammatory cytokines, neurotransmitters production and epigenetic factors such as RNA interference, DNA methylation and histone modification. So, microbiota alteration can produce negative effects on brain function via “gut-brain axis” dysregulation and, indeed, dysbiosis has been reported in many psychiatric conditions^53^ including ASD^47–50^. Recently, the role of mycotoxins has also been proposed in ASD pathogenesis^54,55^. Mycotoxins, worldwide contaminants of food with toxicological effects, induce intestinal permeability and inflammation and interact with gut microbiota resulting in impairment of gut health^56^.

It is well known that the *HP* CNV is not in strong linkage disequilibrium with any individual SNP and therefore it is not successfully genotyped by SNP-array technologies^57^. This is likely due to the fact that *HP1* allele arose from recurrent deletions in *HP2* allele^58^. In addition, *HP1F* and the left copy of *HP2FS* share a 300 bp sequence identical to a segment of Haptoglobin-Related Protein (HPR) gene. This HPR sequence that contains many derived variants was probably transferred into *HP* gene through a paralogous conversion event making *HP* genotyping unpredictable by classic GWAS^58^. Thus, PCR or Real Time-PCR is generally performed for *HP* genotyping on very small cohorts.

Although HP1 allele arose many times, it has been shown that it is possible to impute HP alleles from SNP haplotypes with a high level of accuracy^58^. Indeed, alleles that are old and common today segregate on characteristic SNP haplotypes. In this paper, we assessed the performance of *HP* alleles imputation from SNP haplotypes in our cohort. Overall, we observed a high accuracy in the prediction of *HP* genotypes in both autistic and healthy subjects screened by Illumina and Affymetrix platforms. In both groups, the prediction of *HP*2-2 genotype is more successful (accuracy is 0,97 in both platforms), on the other hand *HP1-1* genotype is lower (0,80 and 0,59, respectively).

Beagle prediction based on the Illumina microarray platform gives acceptable results and should be useful to genotype *HP* for a large scale of subjects. Due to the lower accuracy of *HP1-1* prediction, even if it represents a good accomplishment, a PCR genotyping to confirm *HP1-1* prediction should be useful.

It is important to highlight that the intention of our study was to verify the distribution of *HP2* allele in subjects with ASD and to relate this with GID comorbidity, also driven by the consideration of the increased levels of pre-HP2/zonulin in subject with ASD, measured by ELISA^29^. The unexpected results addressed the question on the possible dissimilarity between zonulin and HP2. During this investigation Scheffler and colleagues^27^ and Ajamian and colleagues^59^ preceded thus we confirm their statements.

## Conclusion

Zonulin is a family of structurally and functionally associated proteins including pre-HP2 and properdin. On the contrary to our expectations, no correlation between *HP* alleles and Italian ASD patients or between subjects with ASD and those patients suffering from GID was found. These results further support a recent study of Schleffer and colleagues^27^. They demonstrated that, within zonulin family members, properdin protein, rather than haptoglobin, is similar to Zot and possibly is involved in intestinal permeability. Interestingly, properdin maps on Xp11.23 show that male subjects are more prone to its effects. Further efforts should be dedicated to genotype and/or to sequencing this gene in subjects with ASD.

However, additional investigations with a wider number of cases and controls are necessarily required to confirm these results. For this purpose, the study proposes a bioinformatics method to predict *HP* allele distribution starting from GWAS data.

Moreover, many issues concerning HP and zonulin definition and detection must still be thoroughly studied. In conjunction with new genetic and/or environmental and predisposing factors which may lead to or provoke leaky gut in autistic subjects.

## Supplementary information

Supplementary table S1.

Supplementary figure S1.
