# Author Correction: Assessment of haptoglobin alleles in autism spectrum disorders

**DOI:** 10.1038/s41598-023-33293-x

**Published:** 2023-04-18

**Authors:** Francesca Anna Cupaioli, Ettore Mosca, Chiara Magri, Massimo Gennarelli, Marco Moscatelli, Maria Elisabetta Raggi, Martina Landini, Nadia Galluccio, Laura Villa, Arianna Bonfanti, Alessandra Renieri, Chiara Fallerini, Alessandra Minelli, Anna Marabotti, Luciano Milanesi, Alessio Fasano, Alessandra Mezzelani

**Affiliations:** 1 Institute of Biomedical Technologies, National Research Council, Via Fratelli Cervi 93, 20090 Segrate, Italy; 2 Department of Molecular and Translational Medicine, Biology and Genetic Unit, University of Brescia, 25123 Brescia, Italy; 3 Genetics Unit, IRCCS Istituto Centro S. Giovanni di Dio, Fatebenefratelli, 25123 Brescia, Italy; 4 Scientific Institute, IRCCS Eugenio Medea, Bosisio Parini, Lecco, Italy; 5 Medical Genetics, University of Siena, Siena, Italy; 6 Genetica Medica, Azienda Ospedaliera Universitaria Senese, Siena, Italy; 7 Department Chemistry and Biology, “A. Zambelli”, University of Salerno, Via Giovanni Paolo II 132, 84084 Fisciano, (SA) Italy; 8 Mucosal Immunology and Biology Research Center, Center for Celiac Research and Treatment, and Division of Pediatric Gastroenterology and Nutrition, Massachusetts General Hospital, Harvard Medical School, 55 Fruit Street, Boston, MA 02114 USA; 9 European Biomedical Research Institute of Salerno (EBRIS), EBRIS Foundation, Via Salvatore de Renzi, 3, 84125 Salerno, Italy

Correction to: *Scientific Reports* 10.1038/s41598-020-64679-w, published online 08 May 2020

The Acknowledgements section in the original version of this Article was incomplete.

“Grants received in support of research work: Italian Ministry of Health (GR-2009-1570296); Italian Flagship-project InterOmics (PB05); European Union’s Horizon 2020 GEMMA project (grant agreement No 825033), www.gemma-project.eu. The Cell line and DNA bank of Rett Syndrome, X-linked mental retardation and other genetic diseases, member of the Telethon Network of Genetic Biobanks (project no. GTB18001), funded by Telethon Italy, and of the EuroBioBank network, provided us with specimens. We thank John Hatton of the Institute of Biomedical Technologies (CNR-ITB) for proofreading the manuscript.”

now reads:

“Grants received in support of research work: Italian Ministry of Health (GR-2009-1570296); Italian Flagship-project InterOmics (PB05); European Union’s Horizon 2020 GEMMA project (grant agreement No 825033), www.gemma-project.eu. The Cell line and DNA bank of Rett Syndrome, X-linked mental retardation and other genetic diseases, member of the Telethon Network of Genetic Biobanks (project no. GTB18001), funded by Telethon Italy, and of the EuroBioBank network, provided us with specimens. We thank John Hatton of the Institute of Biomedical Technologies (CNR-ITB) for proofreading the manuscript. Two of several authors of this publication are members of the European Reference Network for rare malformation syndromes and rare intellectual and neurodevelopmental disorders, ERN-ITHACA.”

The original Article has been corrected.

