# Supplementary material for: Assessment of haptoglobin alleles in autism spectrum disorders
Source: Sci Rep. 2020 May 8;10:7758. doi: 10.1038/s41598-020-64679-w (PMC7210291; doi:10.1038/s41598-020-64679-w)
Supplement: Supplementary file 1 — Supplementary table S1. [file 41598_2020_64679_MOESM1_ESM.docx]

**Assessment of haptoglobin alleles in autism spectrum disorders**

Francesca Anna Cupaioli^1^, Ettore Mosca^1^, Chiara Magri^2^, Massimo Gennarelli^2,3^, Marco Moscatelli^1^, Maria Elisabetta Raggi^4^, Martina Landini^1^, Nadia Galluccio^1^, Laura Villa^4^, Arianna Bonfanti^4^, Alessandra Renieri^5,6^, Chiara Fallerini^5^, Alessandra Minelli^2^, Anna Marabotti^7^, Luciano Milanesi^1^, Alessio Fasano^8,9^, Alessandra Mezzelani^1^*

^1^ Institute for Biomedical Technologies, National Research Council, Via Fratelli Cervi 93, 20090 Segrate (Mi), Italy.

^2^ Department of Molecular and Translational Medicine, Biology and Genetic Unit, University of Brescia, 25123, Brescia, Italy

^3^ Genetics Unit, IRCCS Istituto Centro S. Giovanni di Dio, Fatebenefratelli, 25123, Brescia, Italy.

^4^ Scientific Institute, IRCCS Eugenio Medea, Bosisio Parini, Lecco, Italy

^5^ Medical Genetics, University of Siena, Siena, Italy

^6^ Genetica Medica, Azienda Ospedaliera Universitaria Senese, Siena, Italy

^7^ Dept. Chemistry and Biology, “A. Zambelli”, University of Salerno, Via Giovanni Paolo II 132, 84084 Fisciano (SA), Italy

^8^ Center for Celiac Research, Mucosal Immunology and Biology Research Center and Division of Pediatric Gastroenterology and Nutrition, Massachusetts General Hospital, - East 16th Street, Building 114 (M/S 114-3503) | Charlestown, MA 02114-4404

^9^ Department of Pediatrics, MassGeneral Hospital for Children, 175 Cambridge Street, CPZS – 574 | Boston, MA 02114

* Corresponding author:

Alessandra Mezzelani, PhD

Institute of Biomedical Technologies, National Research Council of Italy; Via Cervi 93, 20090 Segrate (Milan), Italy; Tel: +39 02 26422606; Fax: +39 02 26422660; Email: [alessandra.mezzelani@itb.cnr.it](mailto:alessandra.mezzelani@itb.cnr.it)

**Supplementary Table 1** Sex-based HP allelic distribution in ASDs patients and controls

|  |  |  |  | **Controls enrolled in this study** | | | **Brackenridge, 1971**  **(healthy subjects)** |
| --- | --- | --- | --- | --- | --- | --- | --- |
|  |  |  | **ASD subjects** | **Total** | **Super controls** | **Non-ASD control** |  |
|  |  |  | n=398 | n=379 | n=191 | n=188 | n=1386 |
| **Female** | Genotype | **Hp1-1** | 5 (7.9%) | 18 (8.6%) | 9 (8.8%) | 9 (8.4%) | 94 (13.0%) |
|  |  | **Hp1-2** | 30 (47.6%) | 95 (45.5%) | 44 (43.1%) | 51 (47.7%) | 368 (50.7%) |
|  |  | **Hp2-2** | 28 (44.4%) | 96 (45.9%) | 49 (48.0) | 47 (43.9%) | 263 (36.3%) |
|  | Allele frequency | ***HP1*** | 40 (31.7%) | 131 (31.3%) | 62 (30.4%) | 69 (32.2%) | 556 (38.3%) |
|  |  | ***HP2*** | 86 (68.3%) | 287 (68.7%) | 142 (69.6%) | 45 (67.8%) | 894 (61.7%) |
| **Male** | Genotype | **Hp1-1** | 54 (16.1%) | 17 (10.0%) | 9 (10.1%) | 8 (9.9%) | 103 (15.6%) |
|  |  | **Hp1-2** | 145 (43.3%) | 67 (39.4%) | 24 (27.0%) | 43 (53.1%) | 298 (45.1%) |
|  |  | **Hp2-2** | 136 (40.6%) | 86 (50.6%) | 56 (62.9%) | 30 (37.0%) | 260 (39.3%) |
|  | Allele frequency | ***HP1*** | 253 (37.8%) | 101 (29.7%) | 42 (23.6%) | 59 (36.4%) | 504 (38.1%) |
|  |  | ***HP2*** | 417 (62.2%) | 239 (70.3%) | 136 (76.4%) | 103 (63.6%) | 818 (61.9%) |
|  |  | **P value** | 0.2271 | 0.6354 | 0.1667 | 0.4420 | 0.9066 |
