# Supplementary material for: Assessment of haptoglobin alleles in autism spectrum disorders
Source: Sci Rep. 2020 May 8;10:7758. doi: 10.1038/s41598-020-64679-w (PMC7210291; doi:10.1038/s41598-020-64679-w)
Supplement: Supplementary file 2 — Supplementary figure S1. [file 41598_2020_64679_MOESM2_ESM.docx]

**Assessment of haptoglobin alleles in autism spectrum disorders**

Francesca Anna Cupaioli^1^, Ettore Mosca^1^, Chiara Magri^2^, Massimo Gennarelli^2,3^, Marco Moscatelli^1^, Maria Elisabetta Raggi^4^, Martina Landini^1^, Nadia Galluccio^1^, Laura Villa^4^, Arianna Bonfanti^4^, Alessandra Renieri^5,6^, Chiara Fallerini^5^, Alessandra Minelli^2^, Anna Marabotti^7^, Luciano Milanesi^1^, Alessio Fasano^8,9^, Alessandra Mezzelani^1^*

^1^ Institute for Biomedical Technologies, National Research Council, Via Fratelli Cervi 93, 20090 Segrate (Mi), Italy.

^2^ Department of Molecular and Translational Medicine, Biology and Genetic Unit, University of Brescia, 25123, Brescia, Italy

^3^ Genetics Unit, IRCCS Istituto Centro S. Giovanni di Dio, Fatebenefratelli, 25123, Brescia, Italy.

^4^ Scientific Institute, IRCCS Eugenio Medea, Bosisio Parini, Lecco, Italy

^5^ Medical Genetics, University of Siena, Siena, Italy

^6^ Genetica Medica, Azienda Ospedaliera Universitaria Senese, Siena, Italy

^7^ Dept. Chemistry and Biology, “A. Zambelli”, University of Salerno, Via Giovanni Paolo II 132, 84084 Fisciano (SA), Italy

^8^ Center for Celiac Research, Mucosal Immunology and Biology Research Center and Division of Pediatric Gastroenterology and Nutrition, Massachusetts General Hospital, - East 16th Street, Building 114 (M/S 114-3503) | Charlestown, MA 02114-4404

^9^ Department of Pediatrics, MassGeneral Hospital for Children, 175 Cambridge Street, CPZS – 574 | Boston, MA 02114

* Corresponding author:

Alessandra Mezzelani, PhD

Institute of Biomedical Technologies, National Research Council of Italy; Via Cervi 93, 20090 Segrate (Milan), Italy; Tel: +39 02 26422606; Fax: +39 02 26422660; Email: [alessandra.mezzelani@itb.cnr.it](mailto:alessandra.mezzelani@itb.cnr.it)

**Supplementary Figure 1**

**Supplementary Figure 1** PCR amplicons detected by agarose gel. Full-length gel images. **a**) amplicons obtained by NewA/NewB pair of primers were run on 1% agarose gel. In HP1-1, 1775 bp amplicon was present; in HP1-2, 1775 bp and 3487 bp bands were detected; in HP2-2 carriers only 3487 bp fragment was amplified; **b)** 2% agarose gel was used to detect amplicons by NewC/NewD pair of primers. In HP1-1 genotype the fragment can’t be amplified due to gene structure, and no amplicon was present in the gel; in HP1-2 and HP2-2 genotypes 360 bp amplicons were present.

Images (**c**) and (**d**) correspond to gels in (**a**) and (**b**), and orange rectangulars include gel images in figure 2d and 2e in the paper, respectively.

All images were acquired in monochrome mode with a camera, and have not been processed.
